# Facile Production of Large‐Area Cell Arrays Using Surface‐Assembled Microdroplets

**DOI:** 10.1002/advs.202000769

**Published:** 2020-06-17

**Authors:** Karla Perez‐Toralla, Angel Olivera‐Torres, Mark A. Rose, Amir Monemian Esfahani, Keerthana Reddy, Ruiguo Yang, Stephen A. Morin

**Affiliations:** ^1^ Department of Mechanical and Materials Engineering University of Nebraska‐Lincoln Lincoln NE 68588 USA; ^2^ Department of Chemistry University of Nebraska‐Lincoln Lincoln NE 68588 USA; ^3^ Nebraska Center for Integrated Biomolecular Communication University of Nebraska‐Lincoln Lincoln NE 68588 USA; ^4^ Nebraska Center for Materials and Nanoscience University of Nebraska‐Lincoln Lincoln NE 68588 USA; ^5^ Present address: Laboratoire d'Etudes et de Recherches en Immunoanalyse Université Paris‐Saclay, CEA, INRAE, Département Médicaments et Technologies pour la Santé Gif‐sur‐Yvette 91191 France

**Keywords:** cell arrays, microassembly, microdroplets, surface chemistry, tissue engineering

## Abstract

Techniques that enable the spatial arrangement of living cells into defined patterns are broadly applicable to tissue engineering, drug screening, and cell–cell investigations. Achieving large‐scale patterning with single‐cell resolution while minimizing cell stress/damage is, however, technically challenging using existing methods. Here, a facile and highly scalable technique for the rational design of reconfigurable arrays of cells is reported. Specifically, microdroplets of cell suspensions are assembled using stretchable surface‐chemical patterns which, following incubation, yield ordered arrays of cells. The microdroplets are generated using a microfluidic‐based aerosol spray nozzle that enables control of the volume/size of the droplets delivered to the surface. Assembly of the cell‐loaded microdroplets is achieved via mechanically induced coalescence using substrates with engineered surface‐wettability patterns based on extracellular matrices. Robust cell proliferation inside the patterned areas is demonstrated using standard culture techniques. By combining the scalability of aerosol‐based delivery and microdroplet surface assembly with user‐defined chemical patterns of controlled functionality, the technique reported here provides an innovative methodology for the scalable generation of large‐area cell arrays with flexible geometries and tunable resolution.

## Graphical abstract

Combining microfluidic aerosol generation and stretchable surface‐chemical patterns, a facile method for “micro‐assembly of cells‐in‐droplets,” (µACD) is demonstrated. µACD enables the generation of large‐area cell arrays with tunable geometries and controllable cell loading. This technique has immediate application in cell biology and drug screening with future impacts on the fields of tissue engineering and mechanobiology.

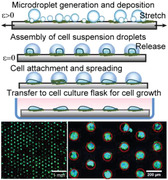

Patterning large‐scale single‐cell arrays enables high‐throughput single‐cell analysis and facilitates in vitro studies of cell–cell communication.^[^ ^1^ ^]^ Several strategies have been developed to create arrays of cells on engineered substrata, including programmed stress fields;^[^ ^2^, ^3^, ^4^, ^5^ ^]^ microfluidic processing;^[^ ^6^, ^7^, ^8^, ^9^, ^10^, ^11^, ^12^ ^]^ surface chemistry, topography, and mechanics;^[^ ^13^, ^14^, ^15^, ^16^, ^17^, ^18^, ^19^ ^]^ and inkjet printing.^[^ ^20^, ^21^, ^22^, ^23^, ^24^, ^25^ ^]^ These techniques provide scientists with the ability to pattern cells for a range of specific applications/studies (e.g., tissue engineering and drug screening), where the appropriate technique is selected based on requirements such as cell count, array resolution, and size. For instance, active patterning techniques, such as the use of optical^[^ ^3^ ^]^ and magnetic tweezers,^[^ ^5^ ^]^ dielectrophoresis,^[^ ^4^ ^]^ or surface acoustic waves,^[^ ^2^ ^]^ can be used to position single cells for the generation of ordered, high‐resolution arrays of cells over small areas.^[^ ^26^ ^]^ Though these techniques require specialized equipment and localized hotspots are possible when external fields are used, the single‐cell resolution and dynamic micromanipulation capabilities offered by these methods are critical to many studies.^[^ ^2^ ^]^ Applications that do not necessarily require single‐cell resolution have benefited from a set of well‐established passive techniques, which rely on the use of micropatterned surfaces with chemical or topographical contrast, such as those generated using photolithography, plasma oxidation,^[^ ^27^ ^]^ or microcontact printing,^[^ ^28^ ^]^ to achieve selective cell attachment, where the size and shape of the cell arrays are controlled at the micro/nanoscale. Here, patterned adsorption of adhesion proteins on mechanically/topographically engineered substrates is realized using customizable microstamps, enabling microcontact‐printing‐based cell patterning over large areas.^[^ ^1^ ^]^ Although these passive methods do not have the same level of dynamic control or reconfigurability as field‐driven methods (a new cycle of photolithography/etching is required to build each unique microstamp, and thus each unique cell array), they have proven extremely useful for applications where scalability and process flexibility are priorities.^[^ ^29^ ^]^ Stencil‐based techniques, which we consider to be a variant of these passive microprinting methods, are similarly useful to applications where scalability and process simplicity are more important than single‐cell resolution. Stencils (which are typically fabricated from thin polymeric membranes) have been used to create custom‐shaped colonies and a variety of cell array configurations,^[^ ^30^, ^31^ ^]^ and much effort has been directed to mitigating unwanted residue on the substrates after stencil removal and to minimizing cell damage during stencil removal.

An alternative set of methodologies, which we draw inspiration from in the present work, make use of droplets to encapsulate cells and pattern cell arrays. Inkjet printing technologies are ideally suited to this approach, and such cell‐loaded microdroplets have been deposited using inkjet printers enabling the generation of cell arrays. Microdroplet‐enabled inkjet cell printing can reach single‐cell resolution,^[^ ^20^, ^21^, ^32^, ^33^ ^]^ though the serial nature of one‐by‐one droplet delivery has introduced new challenges associated with droplet evaporation during the time required to print the array.^[^ ^34^ ^]^ More recently, cell‐loaded droplet arrays have been created using surfaces with super hydrophobic/super hydrophilic contrast patterns where oil was used as a liquid barrier. This method demonstrated the possibility for long‐term cell culture inside droplets and thus the possibility of high‐throughput cell screening based on microdroplet arrays.^[^ ^35^, ^36^, ^37^ ^]^

A 2D cell patterning technique, which combines the dynamic, single‐cell resolution provided by field‐based approaches with the scalable, large‐area arrays accessible using printing‐based methods, would enable a general approach for generating large‐area cell arrays with single‐cell/multi‐cell resolution and customizable array geometries broadly useful to many applications. To achieve such a technique, we sought to combine the advantages of microcontact printing and microdroplet patterning, realizing a new technique for the rational design of cell arrays using microdroplet assembly on stretchable‐chemical patterns for the dynamic assembly (and rearrangement) of individual cells over large areas. To this end, we demonstrated a method for the delivery and assembly of cell‐loaded microdroplets on micropatterned surfaces for the formation of reconfigurable multi‐cell arrays, where consideration of microdroplet deliver/stabilization enabled minimization of harmful perturbations to cell physiology that could compromise meaningful use of the cell array.^[^ ^6^ ^]^ We term this method “micro‐assembly of cells‐in‐droplets” (µACD).

We have previously developed a “surface molding” process that relies on the rational assembly of nebulized microdroplets into ordered arrays using elastomer‐supported surface‐chemical patterns.^[^ ^38^ ^]^ We applied this method to the fabrication of a variety of functional hydrogels and soft actuators, demonstrating the simultaneous formation of more than 20 000 microgels on centimeter‐scale surfaces.^[^ ^39^ ^]^ To expand these capabilities, we have also developed a one‐step approach for the synthesis of chemical patterns with simple, intricate, and reconfigurable geometries on large‐area elastomeric substrates, that are applicable to the manipulation and organization of liquid microdroplets.^[^ ^40^ ^]^ In order to transfer these developments to the assembly of cells‐in‐droplets for generating cell arrays, two technical challenges were addressed: 1) the development of a new strategy for the delivery of millions of cell‐loaded microdroplets that preserved cell viability and 2) the synthesis of specialized stretchable chemical patterns that simultaneously satisfied the need for high wettability contrast to confine and assemble the cell‐loaded microdroplets and the requirement for functional groups that promoted/inhibited cell adhesion and ensured viability of the arrays.

We addressed these challenges, thus enabling µACD, as follows. First, we designed and fabricated a microfluidic aerosol spray technique that minimized shear stress on cells and that was compatible with popular cell media. Second, we demonstrated that stretchable surface chemical patterns constructed using poly‐L‐lysine (PLL, a popular extracellular matrix (ECM)) were able to support surface microdroplet assembly and that these patterns also supported cell viability and proliferation. We can use microfluidic aerosol nozzles to deliver a variety of cells at a range of concentrations simply; we can use stretchable substrata to modulate the geometry of the arrays via mechanical actuation and provide access to a variety of cellular configurations easily. In the future, µACD will make possible the rapid fabrication of cell arrays and, using the same cellular substrata, the application of multiple mechanical/chemical stimuli directly on the cell membrane or at the cell‐substrate interface, paving the way to new applications in single cell screening, tissue engineering and mechanobiology.

In the first critical step of generating cell arrays using microdroplets, we fabricated stretchable chemical patterns using polydimethylsiloxane (PDMS) because of its transparency, biocompatibility, and ease of fabrication and chemical functionalization via plasma oxidation methods. Further, PDMS is an ideal material owed to its mechanical properties that make it suitable for mechanical actuation. We then created an aerosol generator, based on the Venturi easy ambient sonic‐spray ionization (V‐EASI) method,^[^ ^41^, ^42^ ^]^ and applied this tool to the delivery of microdroplets with cell cargo. As compared to other aerosolization techniques (e.g., those based on ultrasound), this method offers several advantages: controlled droplet size, continuous flow stability, low cost, and low energy consumption. It is also easy to assemble (using commercially available parts), and it only requires a high velocity stream of nitrogen or air to operate (e.g., pressurized air cylinders or air compressors), thus offering a potential for portability. Finally, we performed the assembly of A431 cell lines from the human epidermis, a robust organ that routinely undergoes large strains, which are of interest in mechanobiology (e.g., cell–cell and cell–matrix interaction studies)^[^ ^6^, ^29^ ^]^ and are suitable for spray delivery (e.g., in wound care).^[^ ^43^ ^]^

We first modified the reported design of the V‐EASI spraying device to produce a custom apparatus that was suitable for the generation and delivery of cell‐loaded aerosol microdroplets to the assembly substrates. Specifically, we required a system that provided controlled mixing of two fluid phases, an aqueous solution (e.g., a buffer or cell suspension) and a pressurized gas (e.g., air or nitrogen), at the exit of the spraying nozzle (**Figure** **1**A). The effect of the following physical parameters on the control of droplet size distribution, and surface coverage were examined for optimization of the spraying process: flow rate (*Q*), pressure (*P*), nozzle size aperture (*a*), and the nozzle to sample distance (*d*). We used calibrated needles to set the nozzle diameter (*a* = 260 µm) and a moving stage to control the distance (*d* = 15 cm). We used a syringe pump and a pressure regulator to modify independently the applied pressure (*P* = 0–70 kPa) and flow rate (*Q* = 10–100 µL min^−1^) and to achieve the desired spray pattern. In addition, we also evaluated fluid properties (e.g., viscosity, surface tension, vapor pressure, biocompatibility) to enable droplet formation with controlled droplet size and to prevent evaporation. To this end, cell culture media supplemented with HEPES and glycerol was used as cell suspension media during spray to stabilize the pH under atmospheric CO_2_ levels (<0.05%) and to prevent droplet evaporation. To further reduce the risk of liquid evaporation, which could alter the cell media composition and reduce cell viability, the spraying experiment was performed inside a high humidity chamber at >90% relative humidity (RH). We noticed that fluid properties were modified when adding cells to the media, and higher cell concentration led to higher viscosity and lower surface tension,^[^ ^44^ ^]^ which were critical to the size/dispersity of the delivered droplets.

**Figure 1:**
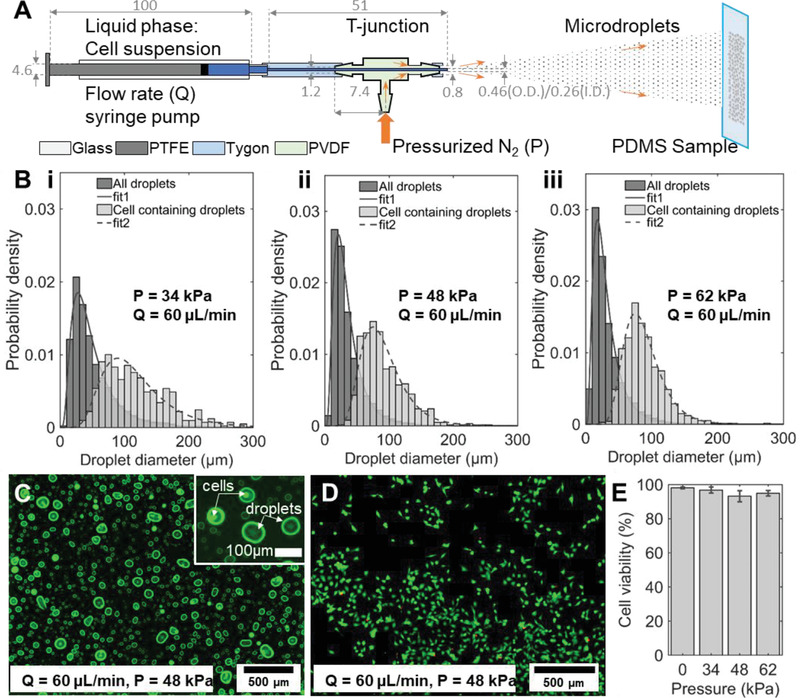
Microfluidic‐based aerosol droplet generation and cells‐in‐droplets deposition. A) Illustration of the droplet generation process including the nozzle design (dimensions in millimeter). B) Size distribution for cell‐loaded microdroplets delivered onto a native PDMS surfaces for different air pressures. The probability distribution for microdroplets with and without cells is given. C) Fluorescence micrograph of microdroplets with cell cargo, sprayed onto native PDMS surfaces (cells labeled with CellTracker green). Inset: magnified view of empty and cell‐loaded droplets. D) Fluorescence micrograph of live/dead assay after cell spay onto plasma‐treated PDMS surfaces (48 kPa) and 24 h of incubation in cell growth media. Live cells labeled with calcein‐AM (green) and dead cells labeled with propidium iodide (red). E) Cell viability measurements for all spraying conditions.

We measured the droplet size distribution after spraying a cell suspension solution onto native hydrophobic PDMS surfaces, using three different pressures (34, 48, and 62 kPa, equivalent of 6, 8, and 10 PSI), and maintaining the other parameters constant (Figure 1B,C; Figure S1, Supporting Information). We observed a decrease in the average droplet diameter and a narrower size distribution for increasing pressures (Figure 1B ‐i to iii). We labeled cells with CellTracker green to differentiate empty droplets from droplets that contained cells on the same surfaces (Figure 1C; Figure S1, Supporting Information). CellTracker is a registered trademark of the company Thermo Fisher Scientific. For samples sprayed with 48 kPa of pressure, even though the average size for the total droplet population is 36 ± 25 µm (mean ± SD), equivalent to 12 ± 4 pL in volume, only droplets above 40 µm in diameter contained cells (Figure 1B ‐ii). This result can be explained by the average size for the A431 cells (10–20 µm) and cell‐triggered Rayleigh−Plateau instabilities during droplet generation.^[^ ^45^ ^]^ We then evaluated the distribution of cell containing droplets and measured an average droplet diameter of 91 ± 35 µm (coefficient of variation (CV) = 38%, equivalent to 197 ± 11 pL in volume) at 48 kPa of pressure. Using higher pressures resulted in narrower size distributions for cell‐containing droplets (88 ± 30 µm, CV = 34%, equivalent to 178 ± 7 pL in volume, for 62 kPa in Figure 1B ‐iii), while lower pressures produced the opposite effect (117 ± 50 µm, CV = 43%, equivalent to 419 ± 33 pL in volume, for 34 kPa in Figure 1B ‐i) (see Supporting Information text and Figure S1, Supporting Information).

To evaluate the effect of shear stress on cell damage during the spraying process, we assessed cell viability 24 h after impact with the PDMS surfaces where the cells were incubated in cell growth media following the spray deposition procedure (at 37 °C and 5% CO_2_). We used a two‐color fluorescence‐based cell viability assay to label and count live cells with calcein‐AM (green) and dead cells with propidium iodide (red) (see Supporting Information text). We measured a viability of 93% ± 3% for 48 kPa of pressure at a flow rate of 60 µL min^−1^ (Figure 1D,E). This viability data remained stable for all spraying conditions (Figure 1E), but was lower than the values obtained with cell delivery via directly pipetting cells to a petri dish (98% ± 1% viability for *P* = 0 kPa, Figure S2, Supporting Information). However, the 93% viability rate is in line with viability data obtained in microfluidic studies.^[^ ^46^ ^]^ This cell survival rate can warrant the technique be applicable to a host of cell types,^[^ ^46^ ^]^ but the technique may still not be gentle enough for some fragile cells such as stem cells or primary cells.^[^ ^47^ ^]^ As expected, higher pressure could lead to the formation of smaller droplets which could increase the resolution of assembly (both in terms of array dimensions and cell count); however, it has been demonstrated that spraying at pressures higher than 100 kPa can significantly increase shear stress during droplet formation and delivery, disrupting cell membrane, and decreasing cell viability.^[^ ^48^, ^49^, ^50^ ^]^ We thus set the operating conditions to *P* = 48 kPa and *Q* = 60 µL min^−1^.

To demonstrate the importance of the composition of the cell suspension media, we also performed cell deposition using 1 wt% sodium alginate solutions in PBS (see Supporting Information). It has been shown that shear thinning properties of alginate can reduce shear stress on cells during droplet formation and alleviate the induced shear upon impact of cells on the substrate;^[^ ^48^, ^51^ ^]^ however, cell viability in alginate droplets was reduced as compared to experiments using cell culture media for the cell suspension (Figure S3, Supporting Information). This effect could be explained by the extended processing times required for the cross‐link of alginate liquid droplets with calcium chloride (100 × 10^−3^ m), which is a diffusion driven process.

In µACD, the generation of an ordered array of cells follows three main steps: 1) the fabrication of an array of micropatterns on the surface of an elastomeric film; 2) the generation and deposition of cell‐loaded aerosol microdroplets; and 3) the assembly of the microdroplets into order arrays for placement of the cell cargo into the desired patterns (**Figure** **2**). We synthesized the required wettability micropatterns by applying O_2_ plasma to PDMS films, through a polymer mask (Figure 2A, step 1), and then immersing the oxidized films in a FITC‐conjugated PLL (FITC‐PLL) solution (Figure 2A, step 2). We rinsed and dried the films to obtain the desired PLL micropatterns for localized cell adhesion (Figure 2A, step 3, and Figure 2B). We observed a preferential adsorption of PLL to the hydrophilic regions (oxidized, hydroxyl‐terminated PDMS) and a limited nonspecific adsorption of PLL to hydrophobic regions (native, methyl‐terminated PDMS).

**Figure 2:**
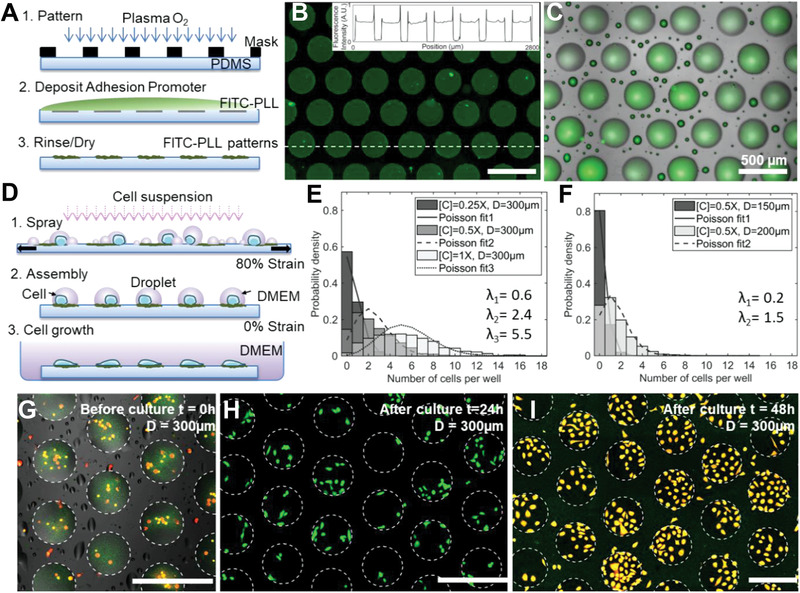
Microassembly of cells‐in‐droplets (µACD) and proliferation of the generated cell arrays. A) Synthesis of wettability micropatterns with cell adhesion promoting matrix on elastomeric films. B) Fluorescence micrographs of FITC‐labeled PLL micropatterns. Inset: fluorescence plot profile across the dotted white line. C) Aqueous droplets (PBS+FITC+10% glycerol) assembled on PLL patterns. D) Procedure for cell/droplet assembly on PDMS‐supported PLL patterns via mechanical actuation. E,F) Probability density of number of cells per well for different pattern sizes and cell concentrations. G) Fluorescence micrograph (300 µm patterns and [C] = 1X) for assembled microdroplets containing living cells (CellTracker orange staining) after strain release and before transferring to cell culture flask. H,I) Fluorescence micrograph of live cells in 300 µm patterns after transfer to cell culture flask and 24 and 48 h of culture for panel (H) (calcein‐AM green staining) and panel I (CellTracker orange staining), respectively. Scale bars = 500 µm.

We performed contact angle measurements (average of 10 water droplets, 1 µL in volume) to quantify the differences in wettability for both the hydrophilic and hydrophobic regions after the PLL functionalization. We obtained a high contrast in wettability when comparing native (*θ* = 114° ± 2°) and plasma‐oxidized (*θ* = 12° ± 5°) PDMS surfaces coated with PLL (50 µg mL^−1^), showing that this fabrication procedure was compatible with the confinement of liquid droplets inside the hydrophilic regions, as required for droplet assembly (Figure 2C).

We then generated cell‐loaded aerosol microdroplets using the procedures described above, while applying a mechanical strain to the functionalized surfaces (*ε* = 80%) (Figure 2D, step 1, and Figure S4, Supporting Information, showing the stretching process). We observed the coalescence and assembly of the generated picoliter droplets on the hydrophilic patterns upon strain release (Figure 2D, step 2), leading to the formation of microscale droplet arrays. The efficiency of the droplet assembly process (i.e., the amount of droplets with the desired volume/size inside the patterns versus the interstitial satellite droplets) has been evaluated previously, demonstrating the importance of the size of the sprayed droplets and the geometrical properties of the patterned arrays (e.g., pattern size, edge‐to‐edge gap, etc.) on assembly efficiency.^[^ ^39^ ^]^ Briefly, the gap between hydrophilic zones was most important to successful assembly and should be approximately three to four times the diameter of the droplets delivered, and more than one strain cycle can be used to collect pinned interstitial droplets during assembly.^[^ ^38^, ^39^ ^]^ Further, these studies demonstrated that post‐assembly strain of the underlying substrate can be used to control the array geometry and basis shape, rationally.^[^ ^38^, ^39^ ^]^ Similarly, spraying and processing times should be kept as short as possible to achieve optimal surface coverage (i.e., uniform distribution of liquid droplets across the patterned area), while preserving cell viability. With the spraying device and operating conditions used in this work (*a* = 260 µm, *d* = 15 cm, *P* = 48 kPa, *Q* = 60 µL min^−1^), we determined a spray delivery time of 60–120 s for surface areas of 1–3 cm² containing circular patterns with 150–300 µm in diameter. Using longer spraying times would result in uncontrolled droplet coalescence on the surfaces, with “over‐filled” patterns and “bridging” defects, deteriorating the quality of the assembled cell arrays. We finally transferred the samples to a cell culture flask and added cell culture media to allow for cell attachment and proliferation (Figure 2D, step 3). We stained cells with CellTracker orange before spraying and observed efficient droplet and cell assembly inside the hydrophilic PLL micropatterns that were labeled in green (FITC staining, Figure 2G), demonstrating the compatibility of this procedure with fluorescence microscopy for real time monitoring of live cells.

We evaluated the distribution of cells inside the assembled liquid droplets (Figure 2E; Figure S5, Supporting Information). We first used a constant pattern size (*D* = 300 µm in diameter) and varied the cell concentration ([C] = 1X, 0.5X, and 0.25X equivalent of 2 million, 1 million, and 500 000 cells mL^–1^). We measured an average of 5 ± 3 cells per well for the highest cell concentration with most wells occupied (>95%), and a broad cell distribution. For the lowest concentration, we obtained 30% of wells with a single cell, but most wells were empty (Figure 2E; Figure S5, Supporting Information), following a Poisson distribution (*λ*_1_ = 0.6). We observed a similar trend when varying the pattern size (diameter *D* = 300, 200, and 150 µm, with solid fraction areas of 0.45, 0.40, and 0.33 respectively) and maintaining the cell concentration constant ([C] = 0.5X, Figure 2F). From our observations of the distribution of droplet size and the distribution in size of cell‐containing droplets (Figure 1B), it is clear that the spray nozzles used deliver many small droplets (<30 µm diameters) that do not contain cells and relatively fewer large droplets (80–100 µm diameters) that contain a cell. The assembly procedure, initiated through release of tension, collects multiple droplets into a single well, where the droplet count is determined by the array geometry (Figure 2D). By tuning the cell concentration, the array geometry, and the nozzle pressure (which we held constant at 48 kPa for assembly studies), we could tune the number of cells per well, rationally (Figure 2E,F).

Following the spray delivery and assembly of cells onto different patterns, we transferred the samples to a cell culture flask and let them proliferate inside an incubator. We observed that viability was preserved (>90%) during the assembly process and that cells were confined inside the patterns after 24–48 h (Figure 2H,I; Figure S6, Supporting Information). We performed the fixation and staining of cells after 24 h of culture (nuclei in blue with Hoechst and actin filaments in green using A‐488‐conjugated phallotoxins) and submitted the samples to fluorescence imaging (**Figure** **3**). We observed a regular array of cells with defined cell colonies inside each pattern (Figure 3A–C). We generated heat color maps (Figure 3D,E) to evaluate the homogeneity of the sample and evaluated the distribution of cells inside each pattern (Figure 3F). We measured an average of 3 ± 2 cells per well for samples with 125 µm patterns and 15 ± 10 cells for samples with 300 µm patterns, with a patterning efficiency higher than 85% (90% of patterns contain cells and 85% of all cells on the sample surface are inside the patterns). The number of cells per well depends on the initial cell concentration and distribution after assembly, as well as on the pattern size and incubation time (number of cell division cycles). Using a lower initial cell concentration, we also observed the attachment, growth, and division of a single cell within one colony for a culture period of 48 h, suggesting that with proper control of cell density, arrays of monoclonal cells can be assembled (Figure 3G). We have focused on the simplest droplet generation/delivery method that results in random cell loading; a more advanced droplet generation procedure (e.g., one based on microfluidic droplet generation) will enable much greater levels of control (in terms of cell count and monodispersity) over cell loading.

**Figure 3:**
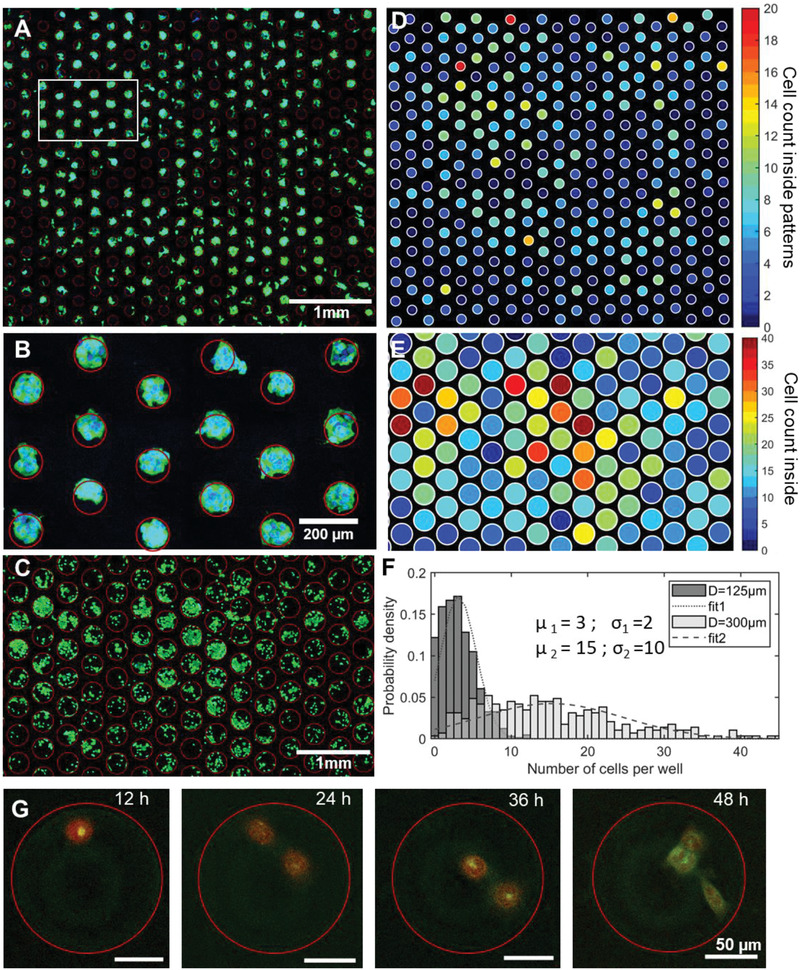
Cell distribution across cell arrays after 24 and 48 h of cell culture. A,B) Inset from A,C) fluorescence micrographs of assembled cells on A,B) 125 µm patterns and C) 300 µm patterns using a cell concentration [C] = 1X. Nuclei labeled with Hoechst (blue) and actin filaments labeled with phallotoxins (green). D,E) Heat map (D: 125 µm patterns, E: 300 µm patterns) for the cell counts using the fluorescence micrographs in (A) and (C). F) Distribution of the number of cells per well for 125 and 300 µm patterns. G) A time‐lapse image series of a single assembled colony with one cell labeled with CellTracker orange cultured for 48 h with observation of every 12 h, when cell attachment, growth and division are clearly visible.

The unique combination of cell‐containing microdroplets and 2D surface patterns in µACD provides opportunities for a wide spectrum of biological applications. Like other droplet‐based methods, drug screening and analytical assays can be conducted right after droplets are assembled and cells in the assembled droplets are in suspended states. Like other 2D patterning methods, tissue engineering and mechanobiology studies are enabled where cells are attached to the substrate and proliferate. Further, besides these monoculture applications, the flexibility of the µACD can be expanded to co‐culture of multiple cells. For instance, in a simple demonstration, we utilized microscale shadow masks and line‐of‐sight deposition procedures to pattern cells in a co‐culture model feasibility study. Specifically, we performed a multi‐step cell deposition procedure using two unique shadow masks and A431 cells labeled with different dyes (CellTracker green and orange) (Figure S7, Supporting Information). We used fluorescence microscopy to validate that the resulting multi‐cell array had the intended patterns (the Nebraska “N” or a series of concentric circles, Figure S7, Supporting Information). In future studies, different types of cells could also be used for cell communication studies (e.g., hepatocytes and fibroblasts). This procedure has the advantage of depositing both types of cells in a single step. Previous demonstrations relied on the use of different surface chemistries to enable preferential cell attachment of one type of cells on a specific area. Other studies used a multi‐step process where the second cell type could only be seeded after the complete proliferation of the first cell type inside the desired pattern.^[^ ^52^ ^]^

We developed a new technique, µACD, for the facile generation of large‐area cell arrays. This procedure relied on the generation of cell‐loaded aerosol microdroplets using a microfluidic‐based spray nozzle and the assembly of these cell‐loaded microdroplets into well‐defined, large‐area arrays using the action of stretchable chemical patterns engineered to include appropriate ECM surface functionality. The procedure we report here combines the advantages of microcontact printing and microdroplet patterning, thus providing a new and versatile route for the generation of cell patterns over large areas with high cell viability and homogeneous coverage. µACD is simple, scalable, and tunable in terms both the array dimensions and the cell population per patterned zone, with the capability of applying uniform mechanical strain along multiple axes for the implementation of mechanosensing assays in future investigations. It can also be adapted to other engineered cellular substrata, to improve the spatial control over surface chemistry (e.g., through the use of multiple markers) and for the deposition of a variety of liquid solutions, cell types, and biological or chemical molecules, for potential applications in drug screening. Although we have mainly demonstrated patterning of 2D surfaces, these tools are transferable for the spray deposition of cells on 3D surfaces useful to the design of scaffolds for tissue engineering. Finally, assembled droplets can also be embedded inside an oil layer or other immiscible fluid for sample partitioning, parallelized bioanalysis, and digital quantification. One of the main challenges for µACD is to ensure the maintenance of cell physiological conditions during the entire delivery and assembly processes. Further studies will establish optimized delivery methods to minimize cell damage from flow‐induced shear stress for better cell survival and to identify matrix proteins with proper surface chemistry for robust cell proliferation. Future developments of µACD will also involve implementing long‐term MTT proliferation assays^[^ ^53^ ^]^ or the use of cell‐based fluid shear stress sensors^[^ ^54^ ^]^ for precise cell health monitoring during the different steps of µACD. Further, like the established methods discussed above, implementation of the strategies and procedures required to use µACD will become increasingly accessible as materials development and automation proceeds. We believe systems that combine electromechanical tensioning devices and pressure‐controlled spray nozzles could make µACD readily accessible in a range of laboratory settings and eventually lead to commercialization. This communication provides an account of the major scientific considerations fundamental to this envisioned outcome and represents a critical first step to future adoption of µACD by other researchers.

## Experimental Section

Further details are provided in the Supporting Information.

## Conflict of Interest

The authors declare no conflict of interest.

## Supporting information

Supporting Information
