# Supplementary material for: Facile Production of Large‐Area Cell Arrays Using Surface‐Assembled Microdroplets
Source: Adv Sci (Weinh). 2020 Jun 17;7(15):2000769. doi: 10.1002/advs.202000769 (PMC7404142; doi:10.1002/advs.202000769)

© 2020 Wiley-VCH GmbH

ADVANCED SCIENCE
Open Access

Supporting Information

for *Adv. Sci.*, DOI: 10.1002/advs.202000769

Facile Production of Large-area Cell Arrays Using Surface-assembled Microdroplets

Karla Perez-Toralla, Angel Olivera-Torres, Mark A. Rose, Amir Monemian Esfahani, Keerthana Reddy, Ruiguo Yang, Stephen A. Morin**

Supporting Information

Facile Production of Large-area Cell Arrays Using Surface-assembled Microdroplets

Karla Perez-Toralla, Angel Olivera-Torres, Mark A. Rose, Amir Monemian Esfahani, Keerthana Reddy, Ruiguo Yang, Stephen A. Morin**

1. Materials and Methods

1.1. Materials

Sylgard® 184 silicone elastomer kit (Dow Corning Co.), acrylonitrile butadiene styrene (ABS-plus) model material (Stratasys Ltd.) for 3D printing were purchased from the noted vendors, and used as received. 2-propanol (CAS# 67-63-0), ethanol (CAS#64-17-5), glycerol (CAS#56-81-5), Triton X-100 (CAS#9002-93-1), Poly-D-Lysine solution, Poly-L-lysine–FITC labeled, sodium alginate and calcium chloride (CAS#10043-52-4) were purchased from Sigma-Aldrich Co. LLC. Gas tight syringes (1001 Model from Hamilton®) and 26 Ga removable needles (51mm length, blunt tip, point style 3) were purchased from Sigma-Aldrich Co. LLC. Masterflex Tygon® tubing (Lab E-3603 L/S 13) was purchased from Cole-Parmer® and Polyethylene Tubing (BTPE-60 by Instech Laboratories, Inc.) was purchased from Fisher Scientific. PVDF barbed plastic Tee connectors (High-Purity, 1/16") were purchased from McMaster-Carr®.

Hanks' Balanced Salt Solution (HBSS), N-2-hydroxyethylpiperazine-N-2-ethane sulfonic acid (HEPES) buffer, Calcein AM, Propidium Iodide, Alexa Fluor™ 488 Phalloidin, Hoechst 33342 trihydrochloride trihydrate, CellTracker™ green CMFDA (5- chloromethylfluorescein diacetate), CellTracker™ orange CMRA and all cell culture reagents were purchased from ThermoFischer Scientific, stored upon arrival and used as indicated by the supplier.

1.2. Methods

1.2.1. PDMS substrate fabrication

We prepared thin PDMS films using a 10:1 base to curing agent ratio, by pouring the degassed prepolymer mixture onto 4-inch silicon wafers (University Wafer Inc.). We used a universal applicator (Zehntner GmbH Testing Instruments, Switzerland) to obtain elastomeric films with a thickness of 500µm. PDMS was cured for 48 hours at 60°C in a convection oven. We cut the prepared films into 30 mm × 6 cm strips and stirred them in ethanol for additional 48 hours at room temperature to remove un-crosslinked monomers, as demonstrated by Regehr et al.[S1] After a rinsing step with DI water, we dried the PDMS strips first under pressurized nitrogen and then inside a vacuum pump for 1 hour to remove ethanol.

1.2.2. Surface functionalization/Micropatterning of elastomeric films

Elastomeric substrates were oxidized in an oxygen plasma chamber (Plasma Etch Inc., Carson City, NV, Model# PE-25 Series) for 10 seconds at a power of 15W, and at ~201.1 mTorr of O_2. Prior to cell deposition, native or oxidized PDMS samples were incubated in PBS for 1 hour at 37°C, rinsed with filtered Millipore water and dried.

For the synthesis of wettability micropatterns, we placed MIMIC masks onto the PDMS surface before the oxidation process.[S2] After removing the masks, we immersed the samples in a PLL solution (50 µg/mL in PBS) for 1 hours at 37°C and rinsed them with Millipore water and dried.

1.2.3. Cell culture

The human epidermoid carcinoma cell line A-431 was obtained as a kind gift from Dr. Kathleen Green's laboratory from Northwestern University. Cells were cultured in Dulbecco's modified Eagle's medium (DMEM) supplemented with 10% fetal bovine serum, and 1% Penicillin Streptomycin (10,000 units mL^{-1}), in 75 cm^2 flask sat 37 °C in a humidified atmosphere with 5% carbon dioxide.

1.2.4. Cell and droplet assembly

1.2.4.1. Sample preparation

Cell lines at 90% confluency were detached from the cell culture flask using trypsin treatment (0.25% Trypsin-EDTA) and resuspended in 5mL DMEM. After a centrifugation (100g, 5 minutes) and a rinsing step with PBS, we discarded the supernatant and re-suspended the cell lines in DMEM supplemented with HEPES (20mM) and 5wt. % Glycerol, pre-warmed at 37°C to a final concentration of 2×10^6 cells/mL.

1.2.4.2. Spraying, aerosol droplet formation and delivery

We loaded the spraying device with 500 µL of cell suspension at secured it on the syringe pump. Cell spraying was performed at 0.01-0.1 mL/min and 0-70 kPa. We set a distance of 15 cm between the spray nozzle and the samples. The samples and spraying device were placed in a humidity chamber, equipped with a humidity sensor (RH> 90%) to prevent the evaporation of microdroplets.

1.2.4.3. Stretching and droplet assembly

We fabricated a stretcher to apply mechanical strain to PDMS strips using solid object printing.[S3] The components were designed in a CAD program (Inventor Professional by Autodesk Inc.) and printed in ABS using a 3D-printer (Dimension Elite, Stratasys Ltd.). (Figure S4). We applied a uniaxial strain by stretching the substrate along its length (L). An increase in the initial length (L) to a final length (L') would provide a strain $\varepsilon = (L'-L)/L$.

1.2.5. Microscopic characterization

Bright field and fluorescence microscopy of the functionalized wettability patterns was performed using a Zeiss microscope (Axio Scope.A1) equipped with a AxioCam MRc5 camera, and the ZEN-Pro software (version 2.3).

1.2.5.1. Fluorescence cell labeling and imaging

To assess cell viability, we labeled living cells with Calcein-AM (0.5 µM, GFP) and dead cells with Propidium Iodide (3µM, TRITC). Cells were incubated in the labeling solution for 30 minutes at 37°C. We then incubated the cells in DMEM at 37°C prior to imaging. We counted the number of live green cells (N_{Live}) and the number of dead red cells (N_{Dead}) for each sample

through fluorescence imaging and obtained: Viability (%) = N_{Live} /(N_{Live}+N_{Dead})*100. A total of 6 samples were performed for each set of spraying conditions during two independent experiments and an average of 2,000 cells were counted for each sample. Cell viability results obtained through this method are reported in Figure 1D and Figure 2H in the main text and Figure S2, S3 and S6.

To monitor the content of sprayed droplets and the spatial distribution of assembled cell arrays on PDMS surfaces, we labeled living cells with CellTracker™ green and orange. These dyes provide bright intensity signals and are retained in live cells for up to 72h, allowing for extended cell monitoring through fluorescence microscopy. Prior to cell spraying and assembly, cells were incubated in the labeling solution (5 µM in serum free DMEM medium) for 30 minutes at 37°C, washed with PBS, suspended in cell culture media supplemented with HEPES+glycerol and loaded inside the spraying device. To prevent photobleaching, cell suspensions labeled with CellTracker™ green and orange dyes were kept away from light. Cells in Figures 1C, 2G, 2I, 3G in the main text and Figures S1, S5 and S7 were stained using this method.

Imaging of the labeled cells was performed using an epi-fluorescence microscope (Nikon Eclipse 80i) equipped with a high speed CCD camera (CoolSNAP HQ2, Photometrics, Roper Scientific - Princeton Instruments), a mercury lamp (HGFIL Lampe 130 W) and adequate filter sets. Fluorescence images for each set of filters were recorded using NIS Elements Imaging Software (Advanced Research, Nikon). We used the ImageJ software (National Institutes of Health, USA) for image analysis.

After 24 or 48h of cell culture, we performed the fluorescence labeling of the actin filaments and the nuclei. Specifically, we washed the cells twice with PBS and fixed them on the PDMS surfaces using formaldehyde solution (4% in PBS) for 10 minutes at room temperature. Following an additional washing step in PBS, we permeabilized the cells with 0.1% Triton X-100 for 3-5 minutes. After washing, we applied 200µL of A-488 conjugated fluorescent phallotoxin solution (5 units/mL) on top of each sample and placed a coverslip for

20 minutes of incubation with the staining solution. Finally, we stained in blue the nuclei with Hoechst solution (1/1000 dilution in PBS) for 10 minutes. Samples were washed and covered with mounting medium and a glass cover slip before confocal imaging (Figures 3 A,B,C in the main text).

1.2.6. Contact angle measurements

Side view photographs for measuring droplet contact angles were taken on a Theta goniometer (Biolin Scientific). An average of 10 nanopore water droplets of 1 µL were deposited on PDMS surfaces with different coatings. Measurements were repeated on three different samples. We used the Young-Laplace equation to fit the shape of each droplet after performing the base line correction and calculated the average contact angle from both sides of the drop.

Supplementary References

[S1] Regehr, K. J.; Domenech, M.; Koepsel, J. T.; Carver, K. C.; Ellison-Zelski, S. J.; Murphy, W. L.; Schuler, L. A.; Alarid, E. T.; Beebe, D. J. Biological Implications of Polydimethylsiloxane-Based Microfluidic Cell Culture. *Lab Chip* **2009**, *9* (15), 2132–2139. https://doi.org/10.1039/b903043c.

[S2] Xia, Y.; Kim, E.; Whitesides, G. M. Micromolding of Polymers in Capillaries: Applications in Microfabrication. *Chem. Mater.* **1996**, *8* (7), 1558–1567. https://doi.org/10.1021/cm9602196.

[S3] McDonald, J. C.; Chabinyc, M. L.; Metallo, S. J.; Anderson, J. R.; Stroock, A. D.; Whitesides, G. M. Prototyping of Microfluidic Devices in Poly(Dimethylsiloxane) Using Solid-Object Printing. *Anal. Chem.* **2002**, *74* (7), 1537–1545. https://doi.org/10.1021/ac010938q.

Supporting Figures

Figure S1: Microscopy image and size distribution and for liquid droplets with cell cargo, sprayed onto native PDMS surfaces using 34kPa and 62kPa of pressure (A, B). Cells are labeled with CellTracker™ Green.

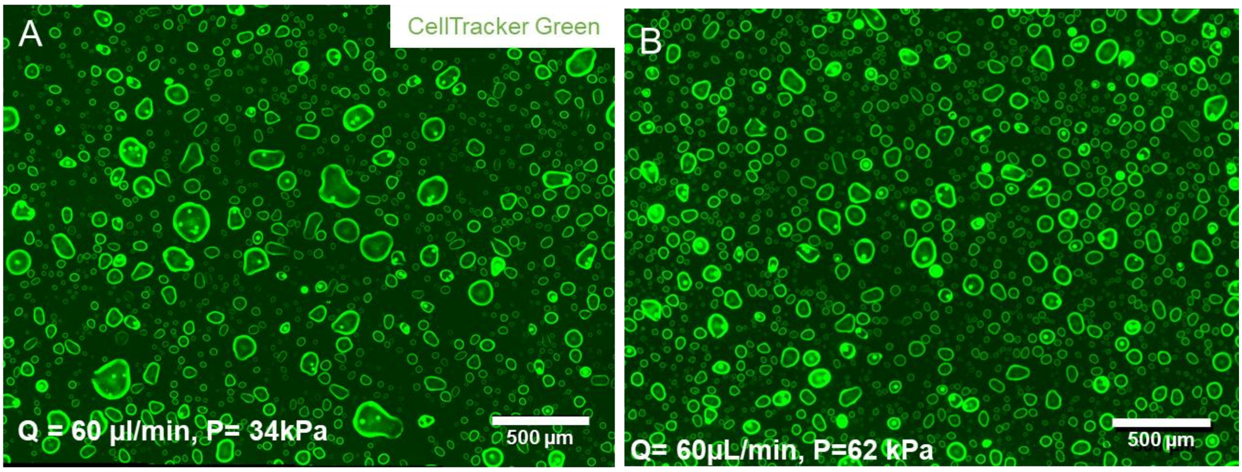

Figure S2: Cell viability after spraying onto oxidized PDMS films for 48 (A) and 62 kPa (B) of pressure, after 24h cell incubation. Cell viability for cells pipetted on petri dish as a reference (0 kPa, C). Live cells are labeled with calcein-AM (green) and dead cells with propidium iodide (red).

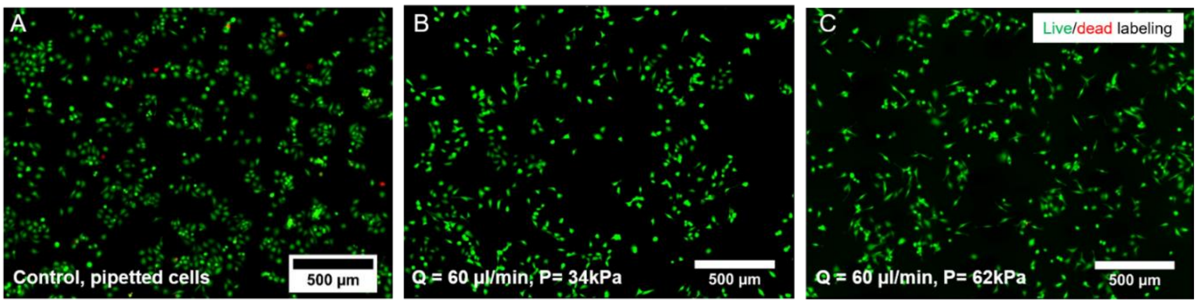

Figure S3: Cell viability after spraying using alginate for the cell suspension media. Cell viability as a function of the sprayed pressure (A). Fluorescence micrographs of cell viability assays for sprayed cells on PDMS using 48 kPa (B) and 62 kPa (C) of pressure, after 24h cell incubation. Live cells are labeled with calcein-AM (green) and dead cells with propidium iodide (red).

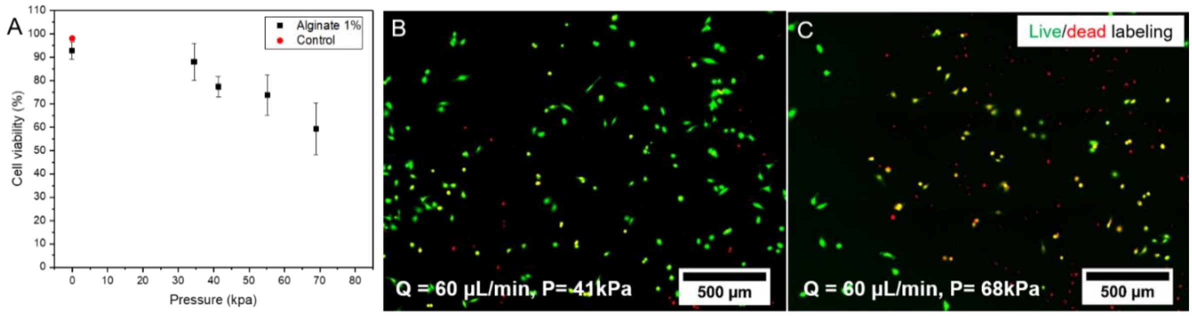

Figure S4. Stretcher device for elastomeric materials made by 3D printing.

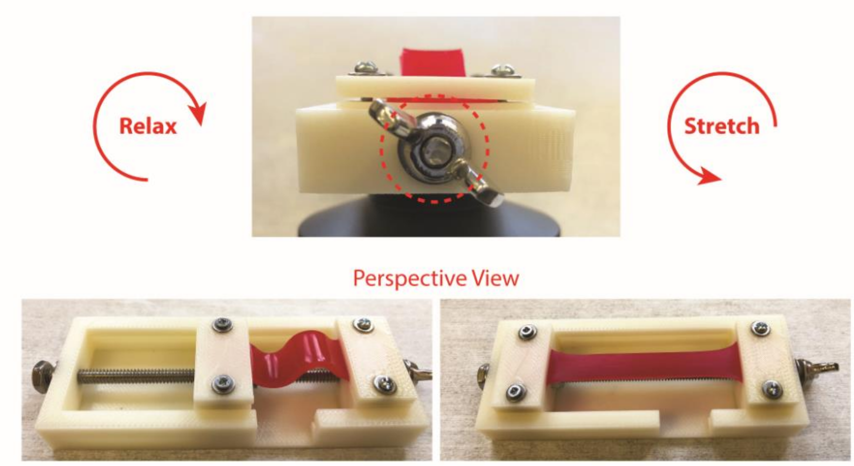

Figure S5. Fluorescence micrographs for the distribution of number of cells per well for 300 µm patterns and for varying cell concentrations [C]=1X (A), [C]=0.5X (B) and [C]=0.25X (C). Patterns are stained with FITC-labeled PLL and cells with CellTracker™ Orange.

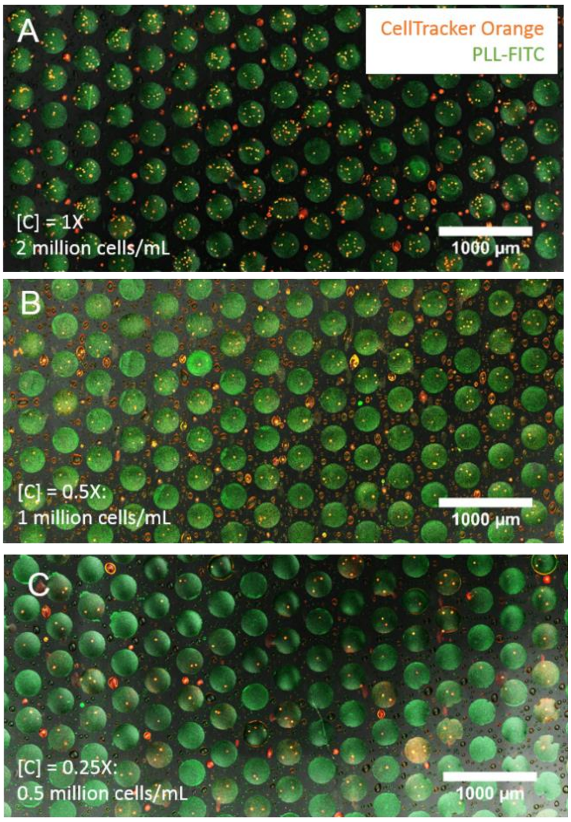

Figure S6: Cell viability after spray delivery and assembly of cells and 24h incubation for different pattern sizes: 150µm (A), 200µm (B). Initial cell concentration [C]=0.5X. Live cells are labeled with calcein-AM (green) and dead cells with propidium iodide (red).

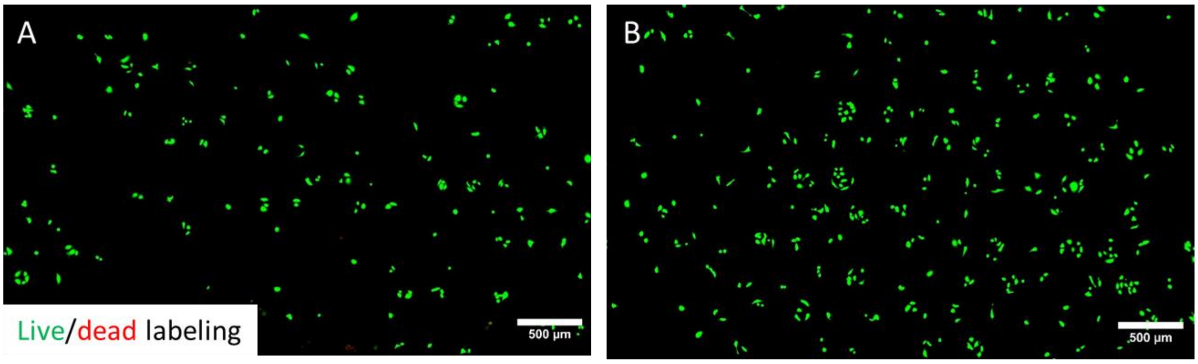

Figure S7: Procedure for multi-cell patterning (A). Two masks are used for the spray deposition and assembly of two types of cells. The pattern of the mask defines the regions for the deposition of each type of cell. Fluorescence micrograph showing the Nebraska "N" pattern (B) and concentric circles (C) with A-431 cells stained with CellTracker™ green or CellTracker™ orange, assembled on elastomeric membranes containing 300µm FITC-labeled PLL patterns. Cross section fluorescence intensity levels for green and red channels extracted from (C).

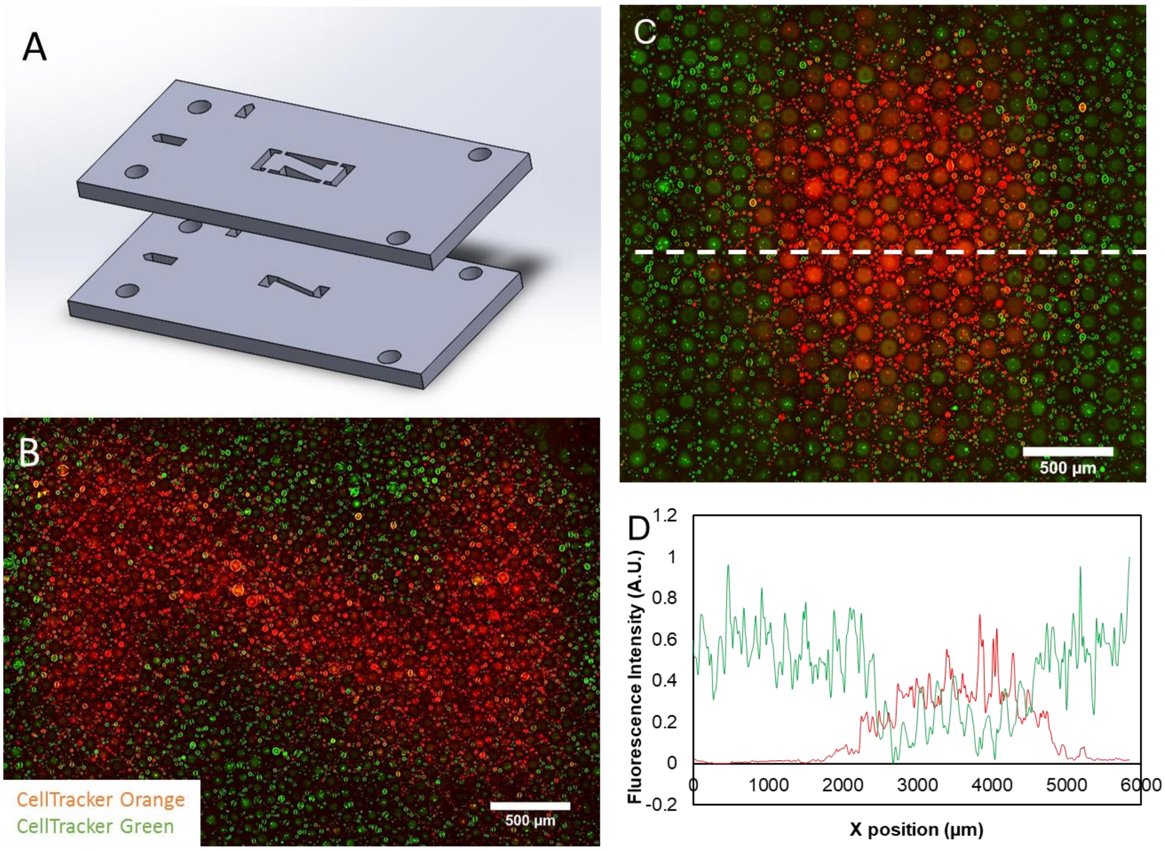

Supplement: Supplementary file 1 — Supporting Information [file ADVS-7-2000769-s001.pdf]
